# Vitamin D and assisted reproductive treatment outcome: a prospective cohort study

**DOI:** 10.1186/s12978-019-0769-7

**Published:** 2019-07-15

**Authors:** Justin Chu, Ioannis Gallos, Aurelio Tobias, Lynne Robinson, Jackson Kirkman-Brown, Rima Dhillon-Smith, Hoda Harb, Abey Eapen, Madhurima Rajkhowa, Arri Coomarasamy

**Affiliations:** 1 Tommy’s National Centre for Miscarriage Research, Institute of Metabolism and Systems Research (IMSR), University of Birmingham, Birmingham, B15 2TT UK; 2 Birmingham Women’s and Children’s National Foundation Trust, Mindelsohn Way, Birmingham, B15 2TG UK; 3 Spanish Council for Scientific Research, Institute of Environmental Assessment and Water Research, Barcelona, Spain; 4 Birmingham Women’s Foundation NHS Trust, Edgbaston, B15 2TG UK

**Keywords:** Vitamin D, Implantation, Assisted reproductive treatment

## Abstract

**Background:**

Vitamin D deficiency has been associated with an increased risk of abnormal pregnancy implantation leading to obstetric complications such as pre-eclampsia and fetal growth restriction. However, the effect of vitamin D on reproductive treatment outcomes in couples undergoing assisted reproductive treatment is poorly understood. This study investigates the association between vitamin D and reproductive treatment outcomes in women undergoing assisted reproductive treatments?

**Methods:**

A prospective cohort study conducted at a large tertiary teaching hospital, United Kingdom. Five hundred women undergoing assisted reproductive treatment were recruited between September 2013 and September 2015. All participants had their serum vitamin D measured and their reproductive treatment outcomes collated. Women were categorised in to three groups: vitamin D replete (> 75 nmol/L), insufficient (50-75 nmol/L) and deficient (< 50 nmol/L) according to Endocrine Society guidance. The primary outcome was live birth. Secondary outcomes included biochemical pregnancy, clinical pregnancy and pregnancy loss rates.

**Results:**

Vitamin D deficiency was found in 53.2% (266/500) of participants and vitamin D insufficiency was found in 30.8% (154/500) of participants. Only 16% (80/500) of women were vitamin D replete. The live birth rates for vitamin D deficient, insufficient and replete women were 23.2% (57/246), 27.0% (38/141) and 37.7% (29/77) respectively (*p* = 0.04). The respective live birth rates for vitamin D deficient, insufficient and replete women were 24.3, 27.1, 34.4% after adjustment for key prognostic factors (*p* = 0.25).

**Conclusions:**

Vitamin D deficiency and insufficiency are common in women undergoing assisted reproductive treatments. The crude live birth rate achieved in women undergoing assisted reproductive treatments are associated with serum vitamin D, although statistical significance is lost when adjusting for important prognostic variables. Vitamin D deficiency could be an important condition to treat in women considering fertility treatment. A research trial to investigate the benefits of vitamin D deficiency treatment would test this hypothesis.

**Trial registration:**

Clinicaltrials.gov - NCT02187146.

**Electronic supplementary material:**

The online version of this article (10.1186/s12978-019-0769-7) contains supplementary material, which is available to authorized users.

## Plain English summary

This study investigates the association between vitamin D and reproductive treatment outcomes in women undergoing assisted reproductive treatments. The study comprises 500 women undergoing in vitro fertilization treatment at a large teaching hospital in the United Kingdom. It is thought that vitamin D is important for the implantation of an embryo to the lining of the womb and deficiency of vitamin D may reduce the chances of pregnancy from in vitro fertilization treatment. Vitamin D is categorized in to three groups; normal vitamin D, insufficient vitamin D and deficient vitamin D. Blood was taken prior to the participants’ treatment and their pregnancy outcomes from their treatments were collated to investigate whether the vitamin status of the women was associated with the rate of implantation, clinical pregnancy, live birth and miscarriage. We found that vitamin D deficiency was common amongst our participants. Furthermore, an association was found between vitamin D status and chances of achieving pregnancy and also live birth. However, when important factors were controlled for, the results lost their statistical significance, although the highest rates of pregnancy were still found in the normal vitamin D group and the lowest rates of pregnancy were found in the deficient group. Vitamin D deficiency could be a condition that could be easily treated in women who are planning to undergo in vitro fertilization treatment for relatively low cost. A trial of vitamin D deficiency treatment should be conducted in women undergoing in vitro fertilization treatment to test this idea.

## Background

Infertility affects one in seven couples in the United Kingdom (UK). In 2014, 52,288 women underwent assisted reproductive treatment (ART) cycles [1]. The overall success rate of these treatments was 36.3% [1]. In vitro fertilisation (IVF) success rates have gradually increased over time due to research in embryology, which has enhanced our abilities to select and transfer the embryo with the highest pregnancy potential [2]. More recently, the rate of improvement in success rates has slowed due to our inability to improve the likelihood of embryo implantation [3].

There has been recent interest in the role of vitamin D in reproductive physiology as vitamin D deficiency has been found to be common in women of resproductive age [4–6]. It is postulated that vitamin D is important in pregnancy implantation as vitamin D enzymes and receptors have been found in the endometrium [7]. Additionally, vitamin D deficiency has been found to reduce fertility capacity in animal studies [8–11]. In humans, the importance of vitamin D in placental function is the most studied aspect of vitamin D in reproduction [12]. Specifically, vitamin D deficiency has been linked to poor placentation and fetal growth restriction [12]. More recently, it has been proposed that vitamin D deficiency leads to improper embryo implantation causing poor placentation [13–15].

The majority of the body’s vitamin D is in the form of vitamin D3 (cholecalciferol), which is photo-chemically synthesised in the skin [16]. Vitamin D levels are usually measured by assay of serum 25-hydroxy vitamin D_3_ concentration. People are at risk of the detrimental effects of vitamin D deficiency at serum 25-hydroxy vitamin D_3_ concentrations of less than 50 nmol/L (less than 20 ng/mL). A level of 50 to 75 nmol/L (21 to 29 ng/mL) is considered insufficient and greater than 75 nmol/L (greater than 30 ng/ml) is considered vitamin D replete [17]. Serum concentrations greater than 374 nmol/L (greater than 150 ng/mL) are associated with toxicity [6, 18–20].

The postulated role of vitamin D in implantation has led research groups to investigate the importance of vitamin D in patients undergoing IVF with conflicting results. None of these studies have been conducted in the UK. In this study, we prospectively examined vitamin D levels of a diverse population of women undergoing ART at an assisted conception unit in Birmingham, UK to identify whether there is an association between serum blood levels of 25-hydroxy vitamin D_3_ and fertility treatment outcomes.

## Methods

The study was funded by the Birmingham Women’s and Children’s NHS Foundation Trust Research and Development Department and was approved by the National Research Ethics Service (NRES) Committee West Midlands – Black Country (REC 13/WM/0258). A total of 504 patients who underwent ART at the Birmingham Women’s Fertility Centre from September 2013 to September 2015 were recruited.

All patients who were referred for IVF, intra-cytoplasmic sperm injection (ICSI) and frozen embryo transfer (FET) treatment met the inclusion criteria and were approached to participate. The only exclusion criterion was that patients were not recruited if they had already participated in the study in a previous IVF treatment cycle. Prospective participants were approached at their treatment consent signing appointment. Vitamin D assay for total 25-hydroxy vitamin D, 25-hydroxy vitamin D_2_ and 25-hydroxy vitamin D_3_ were measured. Assays used a liquid-liquid extraction method using highly sensitive liquid chromatography mass spectrometry (Waters Premier XE MS detector with ACQUITY Ultra Performance LC). Patients were allocated into three groups according to their total 25-hydroxy vitamin D levels; vitamin D deficient group (25-hydroxy vitamin D less than 50 nmol/L), vitamin D insufficient group (25-hydroxy vitamin D 50-75 nmol/L) and vitamin D replete group (25-hydroxy greater than 75 nmol/L) according to the Endocrine Society definitions [17].

### IVF, ICSI and FET treatments

All participants undergoing fresh cycle IVF or ICSI underwent pituitary suppression using either a short antagonist down regulation protocol or a long down regulation protocol using Cetrotide (Merck Serono, France) or Buserelin (Sanofi-Aventis, France) respectively. After complete pituitary suppression, participants underwent ovarian stimulation with recombinant FSH (Gonal-F [Merck Serono, France] or Menopur [Ferring, France]). Starting doses depended on the patient’s age and early follicular phase pituitary follicular stimulating hormone (FSH) level. Doses of exogenous FSH were then modified according to the patient’s ovarian response tracked by transvaginal ultrasonography. The endometrial thickness was also measured at each scan. When at least three follicles reached a diameter of at least 18 mm, 6500 IU of Human Chorionic Gonadotrophin was used to trigger ovulation. In the event of ovarian hyperstimulation or inadequate ovarian stimulation, the treatment was abandoned if clinically required. In women with a suitable ovarian response to exogenous FSH, transvaginal oocyte retrieval was performed 36 h after trigger injection. Morphological grade of all embryos was assessed on days two, three and five post oocyte retrieval. Embryos were graded according to the degree of fragmentation and the regularity of blastomeres. Day five blastocyst grading was according to the development stage, inner cell mass quality and trophectoderm quality. Fresh embryo transfer was conducted in line with the Birmingham Women’s Fertility Centre single embryo replacement policy. Endometrial support was achieved using Gestone injections (Nordic Pharma, France) or Cyclogest pessaries (Actavis, UK) commenced at the time of embryo transfer.

All participants having frozen embryo transfer (FET) treatment underwent pituitary suppression using Buserelin (Sanofi-Aventis, France) followed by oestrogen (Progynova [Bayer, UK] and progesterone administration in the form of Gestone injections (Nordic Pharma, France) or Cyclogest pessaries (Actavis, UK) to prepare the endometrium prior to embryo replacement.

### Outcome measures

The primary outcome was live birth. Secondary reproductive treatment outcomes included biochemical pregnancy rates (positive pregnancy test rates 16 days after oocyte retrieval in fresh IVF or ICSI and two weeks after embryo replacement in FET), clinical pregnancy (presence of a fetal heartbeat on ultrasound scan performed five weeks after embryo replacement) and miscarriage rates. Secondary fertility cycle outcomes included FSH dose requirements, endometrial thickness at last monitoring pelvic ultrasound, number of oocytes retrieved, and quality of embryo/s replaced.

### Statistical analysis

We aimed to recruit 490 patients undergoing IVF treatment (including IVF, ICSI treatment and FET). We hypothesised a pregnancy rate of 35% for women with replete serum vitamin D and 25% for patients with insufficient or deficient serum vitamin D levels. This gives an absolute risk difference of 10%. A 10% attrition rate was anticipated. Aiming for 90% power with a 5% type two error rate, the sample size required was calculated as 490.

The baseline characteristics, ART cycle characteristics, and reproductive treatment outcomes for the vitamin D deficient, insufficient and replete groups were analysed using Mann–Whitney U-tests for non-parametric data and Pearson × 2 tests for categorical data.

Multivariable logistic regression was used to evaluate predictors of live birth, biochemical pregnancy and clinical pregnancy rates. Vitamin D status (deficient, sufficient, replete) was included in the model as an ordinal variable. Variables evaluated as potential confounders included age, body mass index, ethnicity, smoking status, cause of infertility, duration of infertility, baseline pituitary follicular stimulating hormone assay, treatment protocol, and type of treatment. Only those covariates that confounded the relationship between vitamin D and reproductive treatment outcome were included in the adjusted model. Model fit was evaluated using the Hosmer and Lemeshow test. Covariate-adjusted pregnancy and birth rates were estimated using the SPost suite of postestimation commands in Stata. All analyses were conducted using Stata 12.1 (StatCorp.) All *P*-values are two sided, and *P* < .05 was considered statistically significant.

## Results

**Fig. 1:**
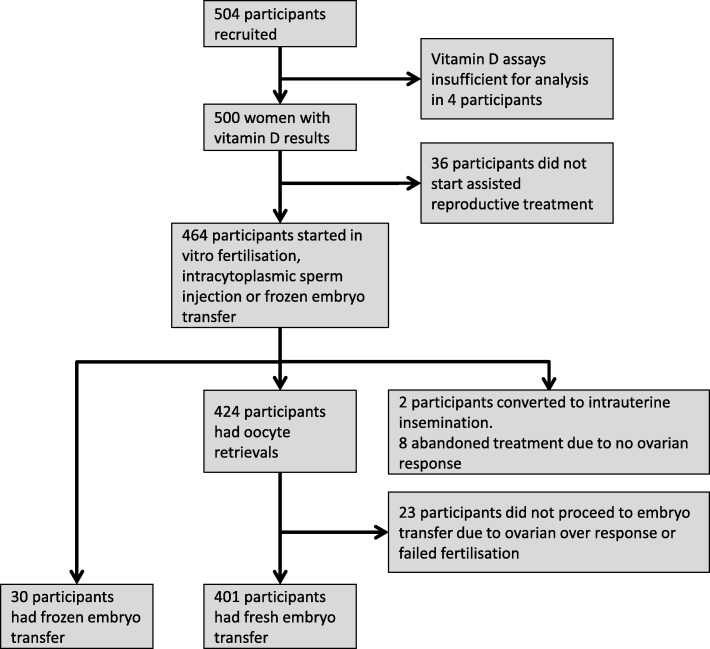
Participant flow chart

The flow of participants through our cohort study is shown in Fig. 1. A total of 504 women were recruited to the study and had serum collected for vitamin D assay. Four blood samples could not be processed for serum vitamin D status due to the blood sample being insufficient. Thirty-six recruited participants never commenced their planned assisted reproductive treatment. The remaining 464 participants commenced their treatment and had their reproductive treatment outcomes collected. Thirty-three participants had cycles abandoned at different stages of their IVF, ICSI and FET treatment either due to ovarian hyper-stimulation, poor ovarian response, failed fertilisation or no oocytes being collected at attempted retrieval. Four hundred and one study participants underwent fresh embryo transfer. Thirty participants underwent a frozen embryo transfer.

### Vitamin D status

**Fig. 2:**
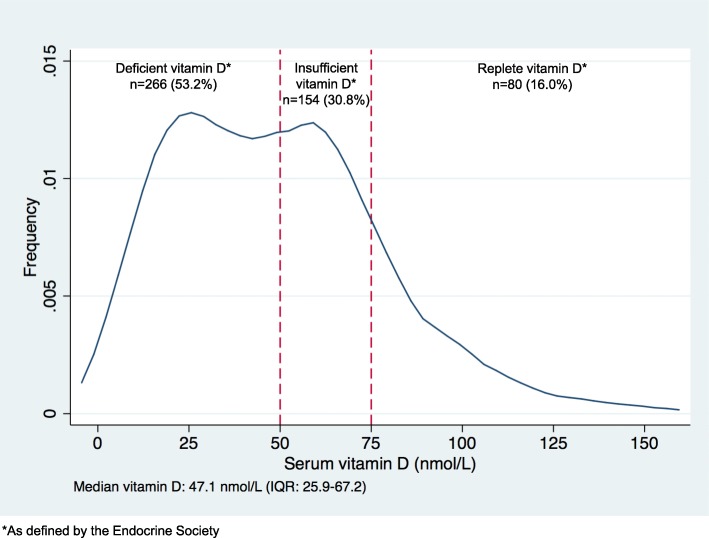
Vitamin D levels for women undergoing assisted reproductive treatments

The vitamin D status of the 500 recruited participants is shown in Fig. 2. Vitamin D deficiency (total vitamin D 0 to 49.9 nmol/L) affected 266 out of 500 (53.2%) participants. Vitamin D insufficiency (total vitamin D 50 to75nmol/L) affected 154 of 500 participants (30.8%). Eighty out of the 500 participants (16.0%) were replete in vitamin D (> 75 nmol/L).

### Baseline characteristics

The baseline characteristics are shown in Table 1. The three vitamin D groups were similar in mean age (*p* = 0.20) and mean body mass index (BMI) for the three vitamin D groups was also comparable (*p* = 0.18). The serum vitamin D levels varied according to ethnicity. More South Asian and Black women were vitamin D deficient, 71.8 and 66.7% respectively. Vitamin D status was more evenly distributed amongst White participants (36.0% deficient, 39.2% insufficient and 24.8% replete). Vitamin D status was unevenly distributed when comparing differing ethnicities and this reached statistical significance (*p* < 0.001). A significant difference was found when comparing vitamin D status with the season. Vitamin D levels were more likely to be in the deficient range in the Winter (59.4%) and Spring (61.6%) when compared to Summer (31.0%) (p < 0.001).

**Table 1:** Baseline characteristics and treatment cycle variables of participants undergoing assisted reproductive treatments

Vitamin D Category				*p*-value
	Deficient^a^ (< 50 nmol/L) *N* = 266	Insufficient^a^ (50-75 nmol/L) *N* = 154	Replete^a^ (> 75 nmol/L) *N* = 80	
Age -years(SD)	33.2 (4.9)	33.7 (4.4)	34.2 (4.6)	0.20
BMI (SD)	24.7 (4.0)	25.1 (3.6)	24.1 (3.1)	0.18
Ethnicity (%)				
White	93 (36.0)	101 (39.2)	64 (24.8)	< 0.001^+^
South Asian	125 (71.8)	37 (21.3)	12 (6.9)	
Black	20 (66.7)	8 (26.7)	2 (6.6)	
Chinese	5 (62.5)	2 (25.0)	1 (12.5)	
Other	23 (76.7)	6 (20.0)	1 (3.3)	
Season (%)				
Winter	76 (59.4)	41 (32.0)	11 (8.6)	< 0.001^+^
Spring	77 (61.6)	39 (31.2)	9 (7.2)	
Summer	31 (31.0)	40 (40.0)	29 (29.0)	
Autumn	82 (55.8)	34 (23.1)	31 (21.1)	
Smoking (%)				
Smokers	16 (6.0)	6 (3.9)	4 (5.0)	0.64^+^
Duration of infertility in months (Median-IQR)	48 (36–72)	36 (24–60)	36 (24–60)	0.04
Mean pituitary FSH (iU/ml) (SD)	7.9 (2.9)	7.6 (2.9)	7.9 (4.0)	0.73
Mean AMH (SD)	18.7 (21.7)	22.6 (27.4)	14.3 (14.1)	0.34
Previous live birth (%)	198 (80.5)	11 (78.7)	62 (80.5)	0.91^+^
Treatment type (%)				
IVF	99 (40.2)	54 (38.3)	40 (52.0)	0.37^+^
ICSI	130 (52.9)	78 (55.3)	33 (42.9)	
FET	17 (6.9)	9 (6.4)	4 (5.1)	
Number of embryos transferred at day 5 post oocyte retrieval (%)	97 (46.6)	65 (53.3)	40 (56.3)	0.28^+^
Number of embryos transferred (%)				
Single	153 (73.6)	79 (64.8)	51 (71.8)	0.23^+^
Double	55 (26.4)	43 (35.2)	20 (28.2)	
Top grade embryo transfer	169 (81.2)	93 (76.2)	56 (78.9)	0.55^+^

^a^As defined by Endocrine Society

^+^*p*-value for chi^2^ test

Smoking is an important prognostic factor in IVF treatment success. Only 26 of the participants smoked. The proportion of smokers in the vitamin D deficient, insufficient and replete groups were comparable (*p* = 0.64).

The key fertility history variables are also displayed in Table 1 and additional information is provided in Additional file 1. Amongst the participants, the duration of infertility was found to be longer in the vitamin D deficient group when compared to the vitamin D insufficient and replete groups. Ovarian reserve, type of treatment, the proportion of women receiving a blastocyst, and the proportion of women undergoing single embryo transfer were similar in all three vitamin D categories.

### Pregnancy outcomes

**Fig. 3:**
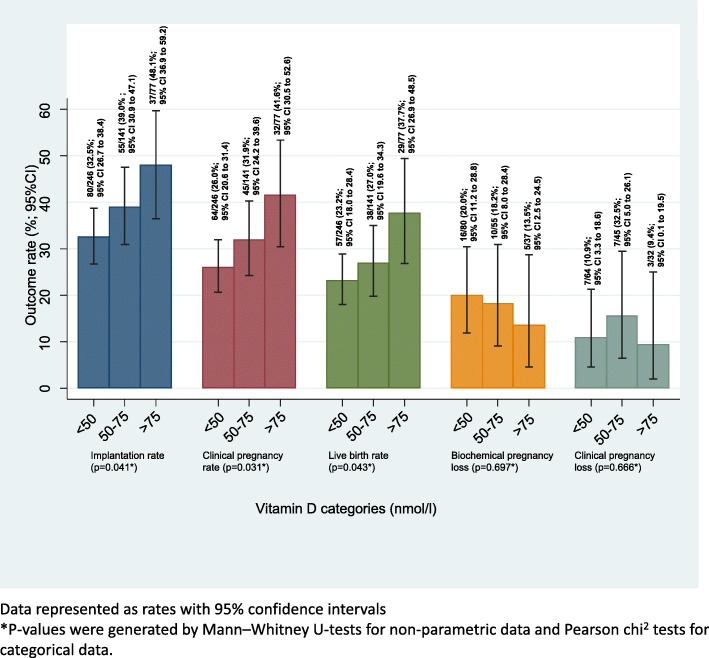
Pregnancy outcomes for women undergoing assisted reproductive treatment by vitamin D levels. Data represented as rates with 95% confidence intervals

The ART outcomes are shown in Table 2 and Fig. 3. Live birth rates in the vitamin D groups were significantly different; the live birth rates in the deficient, insufficient and replete groups were 23.2% (95% CI 18.0 to 28.4), 27.0% (95% CI 19.6 to 34.3) and 37.7% (95% CI 26.9 to 48.5) respectively (*p* = 0.04).

**Table 2:** Outcomes for women undergoing assisted reproductive treatment

		Deficient^a^ < 50 nmol/L n/N % (95% CI)	Insufficient^a^ (50-75 nmol/L) n/N % (95% CI)	Replete^a^ (> 75 nmol/L) n/N % (95% CI)	*p*-value
Live birth	Crude rates	57/246 23.2 (18.0–28.4)	38/141 27.0 (19.6–34.3)	29/77 37.7 (26.9–48.5)	0.04
	Adjusted rates^b^	24.3 (18.7–29.9)	27.1 (19.9–34.2)	34.4 (23.9–44.9)	0.25
Positive pregnancy test	Crude rates	80/246 32.5 (26.7–38.4)	55/141 39.0 (30.9–47.1)	37/77 48.1 (36.9–59.2)	0.04
	Adjusted rates^b^	34.1 (27.9–40.4)	36.7 (28.7–44.8)	43.8 (32.5–55.2)	0.35
Clinical pregnancy	Crude rates	64/246 26.0 (20.6–31.4)	45/141 31.9 (24.2–39.6)	32/77 41.6 (30.5–52.6)	0.03
	Adjusted rates^b^	27.1 (21.2–33.0)	32.0 (24.3–39.7)	38.7 (27.7–49.7)	0.19
Biochemical pregnancy loss	Crude rates	16/80 20.0 (11.2–28.8)	10/55 18.2 (8.0–28.4)	5/37 13.5 (2.5–24.5)	0.70
	Adjusted rates^b^	21.6 (11.6–31.5)	16.1 (5.7–26-5)	14.2 (2.2–26.1)	0.64
Clinical pregnancy loss	Crude rates	7/64 10.9 (3.3–18.6)	7/45 15.6 (5.0–26.1)	3/32 9.4 (0.1–19.5)	0.67
	Adjusted rates^b^	12.2 (4.2–20.2)	22.2 (7.7–36.7)	16.3 (1.8–30.9)	0.55

^a^As defined by Endocrine Society

^b^Adusted for age, body mass index, ethnicity, smoking status, cause of infertility, duration of infertility, baseline pituitary follicular stimulating hormone assay, treatment protocol, and type of treatment

Biochemical pregnancy rates (the percentage of women in each vitamin D group achieving a positive pregnancy test two weeks after embryo transfer) in deficient, insufficient and replete groups were 32.5% (95% CI 26.7 to 38.4), 39.0% (95% CI 30.9 to 47.1) and 48.1% (95% CI 36.9 to 59.2) respectively. The biochemical pregnancy rates show a statistically significant difference between the three groups (*p* = 0.04). Of the women achieving a positive pregnancy test, the biochemical pregnancy loss rates (number of women miscarrying after having a positive pregnancy test and before having fetal heart activity seen on ultrasound scan) for vitamin D deficient, insufficient and replete vitamin groups were 20.0% (95% CI 11.2 to 28.8), 18.2% (95% CI 8.0 to 28.4) and 13.5% (95% CI 2.5 to 24.5) respectively (*p* = 0.70).

The clinical pregnancy rates (the percentage of women achieving fetal heart activity five weeks after embryo transfer) in deficient, insufficient and replete groups were 26.0% (95% CI 20.6 to 31.4), 31.9% (95% CI 24.2 to 39.6), and 41.6% (95% CI 30.5 to 52.6) respectively. The trend in clinical pregnancy between the three groups is statistically significant (*p* = 0.03). Of the women achieving a clinical pregnancy, the clinical pregnancy miscarriage rates for vitamin D deficient, insufficient and replete groups were 10.9% (95% CI 3.3 to 18.6), 15.6% (95% CI 5.0 to 26.1), and 9.4% (95% CI 0.1 to 19.5) respectively (*p* = 0.67).

### The white population

A subgroup analysis of the IVF treatment outcomes in the White population was performed. This was carried out to ascertain whether vitamin D is an independent variable that affects IVF treatment outcome in our largest ethnic group. When comparing vitamin D deficient and insufficient white women with vitamin D replete white women, the live birth rates and clinical pregnancy rates showed clear trends; however, the trend did not reach statistical significance. The live birth rates in vitamin D deficient, insufficient and replete white women were 29.1, 30.8 and 43.6% respectively (*p* = 0.14). The clinical pregnancy rates in vitamin D deficient, insufficient and replete white women were 31.4, 35.2 and 46.8% respectively (*p* = 0.15).

### Multivariate analysis

We performed a multivariate analysis to adjust for several confounding factors. Vitamin D is known to be affected by a patient’s BMI and ethnicity [21]. Smoking, age, cause of infertility, duration of infertility, baseline pituitary FSH assay, type of assisted reproductive treatment performed, and ART protocol used are important prognostic factors associated with pregnancy outcome. The adjusted analysis of crude data from our cohort study shows that live birth, clinical pregnancy and biochemical pregnancy are more likely in women who are replete in vitamin D when compared to women with deficient or insufficient vitamin D levels although the difference in pregnancy outcome rates do not reach statistical significance (Table 2).

## Discussion

### Main findings

Our prospective cohort study indicates that vitamin D deficiency and insufficiency is highly prevalent among women undergoing assisted reproductive treatment. Furthermore, serum vitamin D status is associated with IVF outcomes. Our cohort study is the first to be performed in the UK and the findings confirm what other research groups [22–29] have found in other countries, that women who are vitamin D replete are more likely to achieve a live birth through their IVF treatment than those who are vitamin D deficient or insufficient. When adjusting for confounding factors in our multivariate analysis, trends in reproductive treatment outcomes were maintained, however, statistical significance in live birth, clinical pregnancy and biochemical pregnancy rates was lost. When analysing ART outcomes in the white population (our largest ethnic group), a biological gradient was demonstrated with the highest live birth rates achieved in women replete in vitamin D, and the lowest live birth rates achieved in women deficient in vitamin D.

### Strengths and limitations

There were several strengths to our cohort study. Compared to other similar cohort studies, this study is large. This reduces the likelihood that the association that we found to be due to chance. Additionally, we had no exclusion criteria and aimed to include as many patients undergoing IVF, ICSI and FET treatments as possible. This enables our findings to have greater generalisability. Lastly, the population that we recruited was ethnically diverse, increasing representativeness for the rest of the UK population and specifically all other UK assisted reproductive treatment providers.

The cohort study also had weaknesses. The biological plausibility that vitamin D affects IVF treatment outcome appears to stem from its effects on the endometrium. Ideally, in order to prove the hypothesis that vitamin D exerts its effects on treatment outcome via the endometrium, serum for vitamin D assay should have been obtained on the day of embryo transfer. This ensures that vitamin D status is as close temporally to the time of embryo replacement as possible. In our study, women were approached at the start of their ART. Therefore, there may have been changes and fluctuations to the serum vitamin D level during ART after the assay was performed. However, fluctuations in vitamin D are only achieved when vitamin D deficiency or insufficiency is treated. Although many of the participants may have been taking preconception vitamins at the time the blood test was obtained, the supplementary doses are known to be not high enough to treat and correct vitamin D deficiency or insufficiency [17].

Another weakness in the cohort study is in its observational nature. Although an association between vitamin D and IVF treatment outcome is demonstrated, we are unable to conclude that vitamin D deficiency treatment improves outcomes; only an interventional trial would achieve this.

### Interpretation

Statistical significance was lost when modifying for confounding factors in our multivariate analysis. The confounding factor that led to the loss of statistical significance in our multivariate model was ethnicity. This occurred due to the low numbers of women who were vitamin replete in the non-white ethnic groups. Importantly, despite the loss of statistical significance in our adjusted analysis, the trend of lowest biochemical, clinical and live birth rates in the vitamin D deficient group and the highest biochemical, clinical and live birth rates were maintained.

Interestingly, in our cohort study, when the ART outcomes were analysed in isolation in the white population, women with replete vitamin D levels achieved an increased rate of clinical pregnancy and live birth when compared to those with vitamin D deficiency/insufficiency. Although strong conclusions cannot be drawn from observational data, this would suggest that in white women, vitamin D could play an important independent role in predicting IVF treatment success. This could be via its actions within the endometrium promoting pregnancy implantation or as a surrogate marker for lifestyle and general health.

**Fig. 4:**
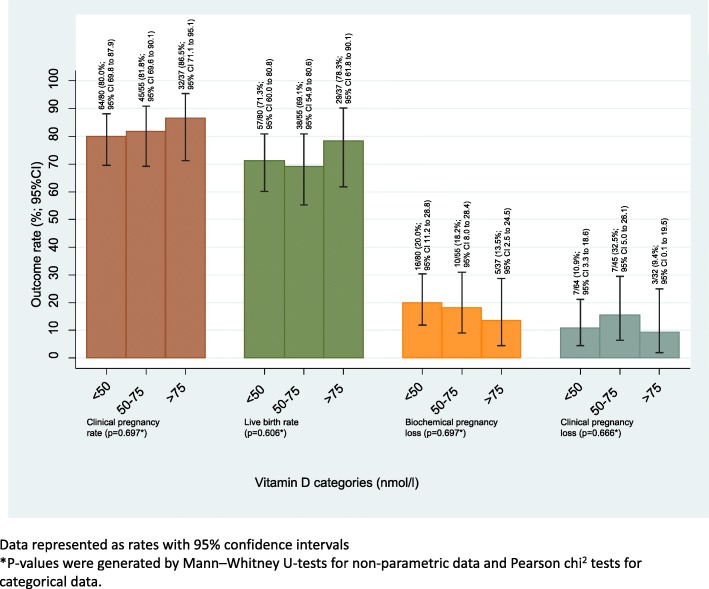
Outcomes for women who achieved a positive pregnancy test with assisted reproductive treatment

In our cohort study, the data shows that if participants are able to achieve implantation (by achieving a positive pregnancy test) the chances of the implanted embryo progressing to become a clinical pregnancy and a live birth are comparable between the three groups (Fig. 4). This supports the theory that vitamin D’s function in fertility is mainly in initial implantation of the embryo.

Seasonal variations in natural conception rates have already been established [30] with higher conception rates found in the Summer and Autumn. Although many hypotheses have been postulated to explain this phenomenon (e.g. reduced ovulation rates and poorer sperm quality in darker months) the exact mechanism behind this has not been explained. It is possible that an increase in sun exposure and greater sunlight luminosity increases the body’s store of vitamin D, thereby yielding higher natural conception rates in Summer and Autumn.

Although the debate regarding the importance of vitamin D and seasonal variation in reproductive health continues, its impact on immunomodulation within the endometrium with a resultant reduction of active inflammatory cytokines is now well understood [16]. The expression of vitamin D receptors at the level of the endometrium and the role of vitamin D in the transcription of HOX10A gene (found to be of key importance in implantation) suggest that the immunomodulatory effects of vitamin D may have a direct impact on implantation and therefore the likelihood of reproductive treatment success.

Other studies have investigated the link between vitamin D status and implantation further. Some research groups have attempted to isolate the effects of vitamin D on the endometrium and implantation by studying women undergoing donor oocyte IVF treatment cycles. In such cycles, one would expect all oocyte prognostic determinants to be nullified, as only high quality oocytes are used. In effect endometrial receptivity and implantation alone are tested. Examples of such studies include the study by Fabris et al., [31] (who did not find an association between vitamin D and implantation in women undergoing IVF treatment using donor oocytes), and the study by Rudick et al., [22] who found a strong association between the vitamin D status of donor oocyte recipients and the IVF treatment success.

In other published research, ethnicity has been found to be a prognostic marker for IVF treatment success on its own [32]. Perhaps the reason for this could be due to higher prevalence of vitamin D deficiency in these ethnic groups due to darker pigmented skin, absorbing a lower level of ultraviolet light in the UK. Consequently, this would reduce the stores of vitamin D in Asian and Black women. Vitamin D receptor gene polymorphisms have already been identified in the Asian population, which may act as a confounding or modifying factor [33, 34].

## Conclusion

In summary, vitamin D deficiency may be a treatable factor that can potentially improve the chances of ART success. Vitamin D serum testing is relatively cheap and widely available. Furthermore, treatment of deficiency or insufficiency with subsequent maintenance therapy is not costly and could also reduce the risk of obstetric complications such as gestational diabetes, pre-eclampsia, and fetal growth restriction. However, an interventional trial may be necessary to establish the effects of vitamin D treatment on assisted reproductive treatment outcomes.

## Additional file

Additional file 1: Additional assisted reproductive treatment cycle variables of cohort study participants. *As defined by Endocrine Society. ^+^*p*-value for chi2 test. (DOCX 59 kb)

## Data Availability

All data generated or analysed during this study are included in this published article [and its additional file].

## Abbreviations

ART: Assisted reproductive treatments
BMI: Body mass index
FET: Frozen embryo transfer
FSH: Follicular stimulating hormone
ICSI: Intra-cytoplasmic sperm injection
IVF: In vitro fertilisation
NRES: National research ethics service
UK: United Kingdom
