# Supplementary material for: Vitamin D and assisted reproductive treatment outcome: a prospective cohort study
Source: Reprod Health. 2019 Jul 15;16:106. doi: 10.1186/s12978-019-0769-7 (PMC6631833; doi:10.1186/s12978-019-0769-7)
Supplement: Supplementary file 1 — Additional assisted reproductive treatment cycle variables of cohort study participants. *As defined by Endocrine Society. +p-value for chi2 test. (DOCX 59 kb) [file 12978_2019_769_MOESM1_ESM.docx]

| **Vitamin D Category** | | | | **p-value** |
| --- | --- | --- | --- | --- |
|  | **Deficient***  **(<50nmol/L)**  **N=266** | **Insufficient***  **(50-75nmol/L)**  **N=154** | **Replete***  **(>75nmol/L)**  **N=80** |  |
| ***Type of infertility (%)*** |  |  |  |  |
| Primary | 136 (55.3) | 81 (57.5) | 39 (50.7) | 0.63⁺ |
| Secondary | 110 (44.7) | 60 (42.5) | 38 (49.3) |  |
| **Cause of infertility (%)** |  |  |  |  |
| Unexplained | 47 (19.0) | 37 (26.2) | 15 (19.5) | 0.77⁺ |
| Anovulatory | 24 (9.7) | 13 (9.2) | 9 (11.7) |  |
| Tubal disease | 39 (15.7) | 16 (11.4) | 15 (19.5) |  |
| Uterine or peritoneal | 15 (6.1) | 6 (4.3) | 5 (6.5) |  |
| Male factor | 91 (36.7) | 51 (36.2) | 21 (27.3) |  |
| Mixed aetiology | 24 (9.8) | 15 (10.6) | 9 (11.7) |  |
| Other cause | 6 (2.4) | 3 (2.1) | 3 (3.9) |  |
| **Treatment protocol (%)** |  |  |  |  |
| Long | 175 (76.4) | 83 (62.9) | 47 (64.4) | 0.04⁺ |
| Flare | 12 (5.3) | 13 (9.9) | 9 (12.3) |  |
| Short | 42 (18.3) | 36 (27.3) | 17 (23.3) |  |
| **Starting FSH dose (%)** |  |  |  |  |
| 150 or less | 97 (42.4) | 55 (41.7) | 28 (38.4) | 0.40⁺ |
| 225-300 | 78 (34.1) | 51 (38.6) | 22 (30.1) |  |
| 300 or more | 54 (23.6) | 26 (19.7) | 23 (31.5) |  |
| **Mean number of FSH ampoules (SD)** | 40.7 (18.7) | 37.4 (19.6) | 42.0 (19.0) | 0.17 |
| **Mean endometrial thickness (mm) (SD)** | 10.9 (2.3) | 10.6 (2.6) | 10.4 (1.8) | 0.24 |
| **Mean number of oocytes retrieved (SD)** | 10.3 (6.6) | 10.4 (5.6) | 9.7 (5.8) | 0.74 |
| **Abandoned cycles (%)** | 37 (15.0) | 18 (12.8) | 6 (7.8) | 0.26⁺ |

**Additional File 1| Additional assisted reproductive treatment cycle variables of cohort study participants**

*As defined by Endocrine Society

⁺p-value for chi^2^ test
